# A protocol for application of OPTIMA-ID to identify potentially inappropriate prescribing in a cohort of older adults with intellectual disability

**DOI:** 10.12688/hrbopenres.14510.1

**Published:** 2026-07-29

**Authors:** Isabel Ryan, Mary McCarron, Philip McCallion, Éilish Burke, Niamh Mulryan, Máire O'Dwyer, Cristín Ryan, Juliette O'Connell

**Affiliations:** 1 Trinity College Dublin School of Pharmacy and Pharmaceutical Sciences, Dublin, Leinster, Ireland; 2 Trinity Centre for Ageing and the Life Course in Intellectual Disability, Trinity College Dublin School of Nursing and Midwifery, Dublin, Leinster, Ireland; 3 Temple University School of Social Work, Philadelphia, Pennsylvania, USA

**Keywords:** intellectual disability, medicines optimisation, pharmacy, ageing

## Abstract

**Background:**

Adults with intellectual disability age differently, with differing comorbid conditions, prescribing patterns, and medication management considerations than the general population. The Optimising Pharmaco-Therapy and Improving Medication for Ageing with Intellectual Disability (OPTIMA-ID) tool is a newly described set of prescribing criteria developed by expert consensus designed specifically for medicines optimisation in older adults with intellectual disability. The Intellectual Disability Supplement to The Irish Longitudinal Study on Ageing (IDS-TILDA) is key source of observational evidence for medicines in this population. OPTIMA-ID must be tested in the population of older adults with intellectual disability to investigate applicability of the tool for medicines optimisation in clinical practice and to describe potentially inappropriate prescribing for the first time in Ireland.

**Methods:**

To design a method for the application of OPTIMA-ID to IDS-TILDA data for the first time, the OPTIMA-ID tool was operationalised by translating the criteria into codable statements. These were used to develop bespoke functions in the programming language R for application to the health, behavioural and medicines data from the fifth Wave of IDS-TILDA data collection.

**Results:**

For the Wave 5 IDS-TILDA dataset, 52 of 67 (78%) OPTIMA-ID criteria were applicable. They were translated into codable statements and built into functions in R for computerised application to the dataset. These functions produce an output of either True or False depending on whether the conditions for each OPTIMA-ID criteria are met, and identify the responsible medicine’s Anatomical Therapeutic Chemical code where relevant.

**Conclusion:**

This protocol describes a method for the application of a novel medicines optimisation tool in a population of older adults with intellectual disability in Ireland, using accurate, computational methods to identify and describe potentially inappropriate prescribing in this population. This will be first study to use OPTIMA-ID to demonstrate its applicability in its intended target population.

## Introduction

### Background

Life expectancy for people with intellectual disabilities has increased greatly due to improvements in medical intervention and the move away from congregated settings. ^1^ ^,^ ^2^ However, inequalities and inequity in health outcomes and morbidity compared to the general ageing population persist, with high rates of preventable causes of death. ^3^ ^,^ ^4^ Older adults with intellectual disability have patterns of disease different to that of the general population. For example, neurological, gastrointestinal and mental health conditions are the most frequently reported diagnoses in older adults with intellectual disability, distinct from the cardiometabolic disease diagnosed more frequently in the general population. ^5^ ^,^ ^6^

Multimorbidity, or having a combination of two or more chronic conditions, is common in this population, reported by 71% and 79.8% of respondents to the Intellectual Disability Supplement to The Irish Longitudinal Study on Ageing (IDS-TILDA) and Healthy Ageing and Intellectual Disabilities (HA-ID) studies, respectively. ^5^ ^,^ ^7^ This has a corresponding effect on the medication burden of older adults with intellectual disability, in particular the high prevalence of antiepileptics, antipsychotics and laxatives prescribed. ^8^ Findings from IDS-TILDA and HA-ID have also revealed greater exposure to polypharmacy (5—9 medicines) and hyperpolypharmacy (10+ medicines) than is observed in older adults in the general population, with researchers reporting polypharmacy at rates between 31.5% and 34.9%. ^8^ ^,^ ^9^ Hyperpolypharmacy (10+ medicines) has been found to be six times more prevalent compared to the general population. ^6^

Long-term treatment with multiple medications can be appropriate in certain chronic conditions such as epilepsy or in multimorbidity, but an increased burden of medicines also increases the risk of prescribing errors and inappropriate prescribing. ^10^ This burden has the potential to cause preventable harm, seen in reported associations with injury, increased dependency, frailty, and mortality in both those with intellectual disability ^9^ ^–^ ^12^ and in the general population. ^13^ ^,^ ^14^ Older adults with intellectual disability are shown to have an elevated sedative burden and anticholinergic cognitive burden, ^15^ which has been associated with reduced functional independence ^16^ and an increased risk of falls with exposure over time. ^17^

This population can also experience age-related conditions and symptoms of age-related decline earlier than the general population, such as higher incidence of Alzheimer’s disease in individuals with Down’s syndrome, ^18^ degenerative arthritis, osteoporosis, and early menopause. ^5^ ^,^ ^19^ ^–^ ^21^ Life expectancy is on average nineteen years shorter than that of the general population ^4^ and consequently, older age is often redefined in this cohort, e.g. as ≥40 years. ^5^ It is hypothesised that differences in the ageing process in adults with intellectual disability may impact drug pharmacodynamics and pharmacokinetics earlier and in a different manner to the general population, due to differences in body fat distribution, expression of metabolic enzymes, genetic abnormalities and neurological differences. ^22^ Increased sensitivity to drug adverse effects, such as movement disorders as a side effect of certain antipsychotics ^23^ and drug-drug interactions is compounded by the high prevalence of antipsychotics, often prescribed for behaviours that challenge without a clinical diagnosis of psychosis or other appropriate indication. This off-label use has little evidence supporting its safety or efficacy. ^24^ ^–^ ^26^

Management of comorbid conditions, the resulting polypharmacy, and the additional considerations for prescribing in older adults with intellectual disabilities signal the importance of medicines optimisation in this population. ^27^ Medicines optimisation promotes the safe and effective use of medicines through medicine reconciliation and review, enabling rational medicines use and desirable health outcomes for people taking one or more medicines. ^28^ Medication review by prescribers and pharmacists with interdisciplinary input can identify potentially inappropriate prescribing (PIP); prescribing that could cause or contribute to medicines-related harm, particularly in polypharmacy or comorbid conditions. ^29^ ^–^ ^31^ PIP encompasses potentially inappropriate medicines which may include drug-drug interactions, non-evidence-based therapies, and medicines considered high-risk in older people; and potential prescribing omissions, the lack of appropriate treatment despite a clear indication.

In the general population, prescribing tools for the identification of PIP such as Beer’s Criteria for potentially inappropriate medication use in older adults, ^32^ the Screening Tool of Older Person’s Prescriptions (STOPP), and the Screening Tool to Alert doctors to Right Treatment (START) ^33^ have been shown to predict hospitalisation and functional decline. ^34^ These explicit prescribing guidelines provide opportunities to make improvements to prescribing for older adults. However, because of differences in morbidity and prescribing patterns, they may not adequately address the needs of older adults with intellectual disability. ^35^ ^–^ ^37^ Older adults with intellectual disabilities are also sometimes faced with difficulties in communication, prescribing cascades, and diagnostic overshadowing, all of which can be barriers to medicines optimisation. ^35^ ^,^ ^36^ ^,^ ^38^

The Optimising Pharmaco-Therapy and Improving Medication for Ageing with Intellectual Disability (OPTIMA-ID) screening tool was designed to provide the evidence-based guidance needed to support medicines optimisation in older adults with intellectual disability. ^39^ It was validated by Delphi consensus. ^39^ ^,^ ^40^ Focus groups of experts in prescribing for adults with intellectual disability and experts by lived experience of intellectual disability contributed to its development. The tool is comprised of 67 criteria across eight therapeutic areas: general principles of pharmacotherapy, alimentary tract and metabolism, cardiovascular system, genitourinary system and sex hormones, systemic hormonal preparations, musculoskeletal system, nervous system, and respiratory system. ^39^ It also incorporates other pre-existing tools for optimising prescribing, including the Anticholinergic Cognitive Burden (ACB) scale. ^41^

As a set of explicit prescribing, screening and non-pharmacological criteria, OPTIMA-ID aims to strike a balance between representing the complexity of medication use in this population, focusing on a pragmatic portion of the most prevalent medication and disease classes found in older adults with intellectual disability. This meant the exclusion of less relevant or redundant criteria commonly found in optimisation tools for the general population to keep focus on the domains requiring the most attention in older adults with intellectual disabilities. ^39^ The criteria identify potentially inappropriate medicines and potential prescribing omissions, but also non-pharmacological issues, such as lack of non-pharmacological intervention, monitoring or patient-centred counselling. ^39^

OPTIMA-ID is intended for use by specialists and non-specialists, providing a guide through the rapidly growing evidence base surrounding health management in older adults with intellectual disability as the proportion of people with intellectual disability living into old age increases. ^1^ ^,^ ^2^ With the move of adults with intellectual disability out of congregated settings and into the community in Ireland and internationally, ^42^ ^–^ ^45^ community healthcare professionals are encountering this growing population more in their practice and need support and education for their complex health management needs. ^35^ ^,^ ^36^ ^,^ ^38^ OPTIMA-ID aims to support general practitioners (GP), pharmacists and other health professionals in identifying PIP and engaging with medicines optimisation in older adults with intellectual disabilities. ^39^

Despite findings supporting regular medication review for people with intellectual disabilities, ^46^ they remain uncommon, and the prevalence of PIP has not been extensively studied, due in part to a lack of specific screening tools and deprescribing protocols. ^35^ Some studies have identified inappropriate prescribing and prescription errors in adults with intellectual disability using medication review and audit, including in a nationally representative study in the Netherlands. ^10^ ^,^ ^47^ ^–^ ^49^ Other medication review tools and prescribing aids developed specifically for adults with intellectual disability include the Tool for Optimising Prescription in Intellectual Disability (TOP-ID) ^50^ and the Tool for Appropriate Psychotropic Drug Prescribing in people with Intellectual Disabilities (TAPP-ID). ^51^ A Systematic Tool to Reduce Inappropriate Prescribing (STRIP), which is an adapted version of the STOPP/START tool, has also been piloted for the identification of PIP in adults with intellectual disability. ^47^ Unlike OPTIMA-ID, these tools are either not catered specifically to the age-related medication issues of older adults with intellectual disability or focus specifically on psychotropic drug optimisation. OPTIMA-ID includes drug-specific, explicit issues of particular concern based on expert consensus, including in non-neurological, non-psychiatric therapeutic domains. ^39^

In order to continue the development of OPTIMA-ID as a medicines optimisation tool, its applicability to the population must be evaluated to support the interpretation of the tool as a reflection of PIP in older adults with intellectual disability. ^39^ This will be the first time PIP will be identified with application of OPTIMA-ID to a representative population of older adults with intellectual disability, the first using data from the IDS-TILDA, and therefore the first in the Irish population, to holistically assess self-reported medication use with an explicit prescribing tool.

IDS-TILDA has completed five waves of data collection (Wave 1, 2010—2011; Wave 2, 2013—2014; Wave 3, 2016—2017; Wave 4, 2019—2020; Wave 5, 2022—2023), with Wave 6 in progress (2025—2026). The most prevalent OPTIMA-ID criteria in the cohort at the most recent points of data collection, Waves 5 and 6, will be identified. Associations between PIP and reported demographics and healthcare utilisation will be investigated, contributing to the topic of medicines optimisation for older adults with intellectual disability, which is rarely-researched and in need of investigation. ^35^

### Objectives

The aim of this study is to evaluate the prevalence of PIP in older adults with intellectual disability in Ireland and to explore associations between PIP and health outcomes in this group. Data from Wave 5 will provide a current reflection of the self-reported medicines use in the Irish cohort of older adults with intellectual disabilities. The objectives of this study are to:

1. Apply the OPTIMA-ID criteria to existing medication and health data of older adults with intellectual disability in IDS-TILDA.
2. Determine the prevalence of PIP in the Wave 5 IDS-TILDA cohort and identify the most common instances of PIP.
3. Examine association between PIP and demographics, polypharmacy, multimorbidity and healthcare utilisation at Wave 5, adjusting for confounding variables.
4. Examine association between PIP and healthcare utilisation, functional decline and survival at Wave 6, collected three years following Wave 5, adjusting for confounding variables.

## Methods

### Study design

This retrospective cohort study will identify potentially inappropriate prescribing using the OPTIMA-ID tool, in a cohort of older adults with intellectual disability participating in the IDS-TILDA study. It will focus on the most recent Waves of data collection, i.e. Waves 5 and 6, between the years 2022—2023 and 2025—2026, in order to capture a current picture of prescribing practice in this population. A total of 762 individuals participated in Wave 5, with Wave 6 currently in progress.

### Sources of data

IDS-TILDA is an Irish longitudinal study that follows a representative sample of adults with intellectual disability aged ≥40 years from whom data are collected through a Pre-Interview Questionnaire (PIQ), completed by the participant and/or by a proxy, and a Computer-Assisted Personal Interview (CAPI). The PIQ collects information about prescribed medication, behaviours that challenge, healthcare utilisation, and routing screening tests. The CAPI is an extensive personal interview which collects data about medical history, mental health, and social participation. Different interview styles are employed depending on the level of intellectual disability and communication abilities of the participant, including interview with support from a proxy (a person involved in caring for the participant who has known them for at least six months), interview independent of proxy, or proxy-only interview. Medication data recorded in the PIQ are verified during the CAPI by confirming with the respondent or their proxy or cross-referencing against the respondent’s medication Kardex, where possible. Medication data captured include medicine name, dosage, frequency, route of administration, and date first prescribed. These data are transcribed and the World Health Organisation Anatomical Therapeutic Chemical (ATC) code and international non-proprietary name (INN) are added for each entry. Medicines data are verified by a research pharmacist.

**Figure 1.**
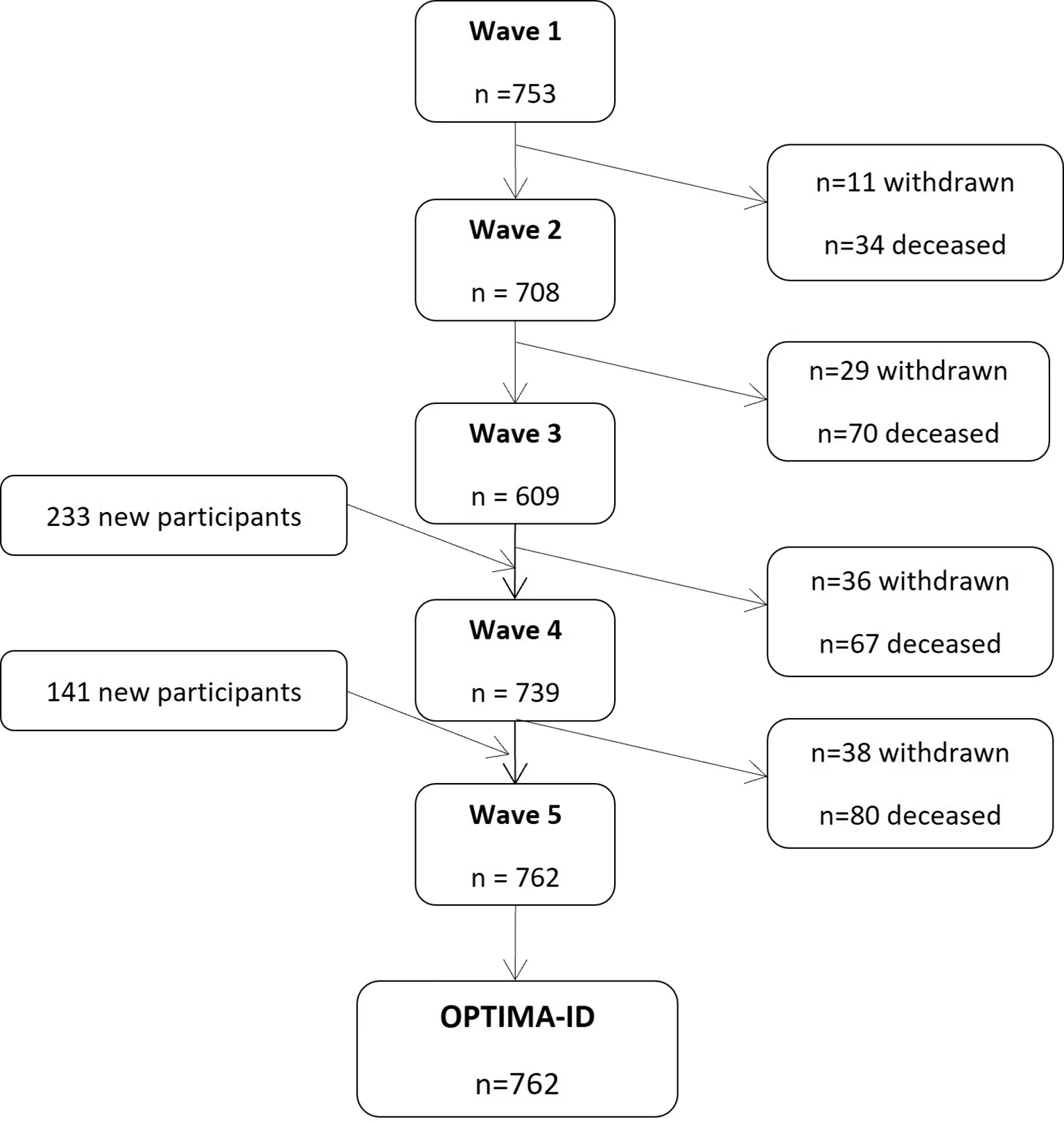
IDS-TILDA study participation Wave 1 to Wave 5.

Ethical approval for Waves 5 and 6 were secured in 2022 and 2024 respectively, from Trinity College Dublin Faculty of Health Sciences Ethics Committee and organisations supporting people with intellectual disability. Participant retention throughout the IDS-TILDA from Wave 1 to Wave 5 is presented in Figure 1.

### Population

The initial IDS-TILDA sample was randomly selected from the National Intellectual Disability Database, now known as the National Ability Supports System. The sample was refreshed prior to Wave 4 (233 new participants) and Wave 5 (141 new participants) to account for the ageing of the participants in the initial sample, withdrawal or death. Participants with any level of intellectual disability and the full range of living circumstances, including residential (living arrangements where ≥10 share a single living unit or are campus-based), community group home (community setting with staff support for small groups) or independent/with family, were included.

Participants were recruited to be nationally representative of the Irish population of adults ≥40 years with intellectual disability. The inclusion of adults ≥40 years reflects the reduced life expectancy and premature ageing common in this population. ^4^ ^,^ ^5^ Written consent to participate from each participant or a written agreement from the participant’s family/guardian was obtained before commencement of the study. This consent is reaffirmed for existing participants by interviewers at each Wave of data collection. Due care and consideration is taken by interviewers considering the vulnerability of the study population.

### Data operationalisation

Criteria will be applied to the health and medicines data using bespoke functions built using R Statistical Software. ^52^ Out of 67 OPTIMA-ID criteria, which span eight therapeutic domains, sufficient data are available to design functions to apply 52 criteria (78%) to participants from Wave 5 of IDS-TILDA.

The genitourinary system/sex hormones (n = 3, 100%), systemic hormonal preparations (n = 2, 100%), and the musculoskeletal system (n = 2, 100%) are the domains with sufficient data to apply all criteria therein, followed by the alimentary tract and metabolism (n = 14, 93%), nervous system (n = 19, 86%), cardiovascular system (n = 7, 78%) and respiratory system (n = 2, 67%). General principles of pharmacotherapy has the fewest applicable criteria (n = 4, 36%); it largely pertains to medication review, multidisciplinary review, and deprescribing, and would require access to more extensive patient medical history or medical team notes in order to assess, which were not collected at Wave 5 of IDS-TILDA.

In order to map the OPTIMA-ID criteria to Wave 5 IDS-TILDA data, rules were assigned to each criterion and developed into codable statements, described in **Extended Data 1.** ^53^ Where it was not possible to apply a criterion to the Wave 5 dataset, a rationale is provided. These rules were coded as functions to be applied to the gathered data, which produce a set of dichotomous variables indicating if the conditions for each criterion have been met (True) or not (False). Full detail of the R code used can be found in **Extended Data 2.** ^53^ The functions also identify the ATC code of the medication responsible for triggering the criterion. Participants for whom the relevant information is unknown, for example, if medication data are missing, are recorded as Not Applicable (NA).

### Data analysis

The prevalence of OPTIMA-ID criteria in the sample population will be reported, along with participant demographic characteristics including sex, age, level of intellectual disability (mild, moderate, severe/profound), and residence (community group home, residential, independent/with family). Other health-related variables such as multimorbidity, defined using 12 chronic health conditions as per previous studies in this cohort, ^5^ a modified Barthel Index as a measure of functional dependence in activities of daily living ^16^ ^,^ ^54^ and polypharmacy will also be reported. Descriptive statistics for medicines that are triggering the most criteria and the number and type of potentially inappropriate medicines per participant may also be reported. Participants who did not report any medicines data will not be excluded from descriptive statistics so as not to exclude those with any non-pharmacological issues captured by the OPTIMA-ID tool.

Polypharmacy will be calculated by summing all regular medicines participants reported when asked to record “… all medications that you take on a regular basis, every day or every week.”, excluding supplements (omega-3 triglycerides [C10AX06], glucosamine [M01AX05], calcium [A12AA], B vitamins [A11D, A11E, B03B], iron [B03A], multivitamins with or without minerals [A11A, A11B], vitamin C [A11GA01], or vitamin D or its analogues [A11CC]) and medical devices. ^6^ Participants are considered to have polypharmacy if they report taking five to nine medicines, and hyperpolypharmacy if they report taking ten or more.

Statistical analyses will be carried out separately for having one or more of any OPTIMA-ID criteria, and for having one or more instances of PIP specifically. Consideration of PIP-specific criteria as a subset of all OPTIMA-ID criteria will allow for medication-specific analysis and potential comparison with associations found using other prescribing tools.

Cross-sectional association between OPTIMA-ID criteria/PIP and demographics, polypharmacy, or multimorbidity at Wave 5 will be investigated, followed by longitudinal association with OPTIMA-ID criteria/PIP at Wave 5 and health data at Wave 6 (three-year follow-up). Confounding variables will be identified by investigating the statistical significance of bivariate association with significance assumed at *p < 0.05.* These will be used to adjust multivariate regression models investigating the odds ratio for OPTIMA-ID criteria/PIP with health variables which may include falls, reduction in Barthel Index and healthcare utilisation (GP visits, hospitalisations, nights in hospital, emergency department visits, and outpatient visits) between Wave 5 and Wave 6. Those who did not report medicines data or with an unknown level of intellectual disability will be excluded from regression analyses as in previous studies in this population. ^8^ ^,^ ^55^

Modelling decisions will be made depending on the response variable of interest e.g. logistic regression, negative binomial regression and additional models may be considered for specific therapeutic domains or individual OPTIMA-ID criteria. Appropriateness of the sample size (total n = 762) for multivariate regression will be determined using Peduzzi’s rule of thumb of ten cases per independent variable. ^56^ Multicollinearity will be assessed using Spearman’s rho and Variance Inflation Factor. In consultation with the IDS-TILDA research team, reporting of datapoints may be limited so as to safeguard the identity of the study participants.

### Dissemination

The findings from this study are intended to be published in a pharmacy, pharmacoepidemiology, intellectual disability or other appropriate research journal. It will also be presented in an accessible manner to individuals with intellectual disabilities, which may include images or diagrams with straightforward, lay explanations, including the potential impact of improving prescribing and health outcomes on individuals with intellectual disabilities, with an adaptable approach taken where needed. The Trinity Centre for Ageing and the Life Course in Intellectual Disabilities’ Public Participant Involvement panel will also be consulted.

This is to uphold the core tenet “nothing about us without us” that is crucial to the workings of the IDS-TILDA, keeping adults with intellectual disabilities informed about current research regarding their present and future, and taking direction from their lived experience. There will also be engagement with healthcare practitioners working with older adults with intellectual disabilities, or who may encounter them in their clinical practice, to encourage the use of this prescribing aid for medicines optimisation in this group, once applicability has been demonstrated.

## Discussion

### Strengths

This is the first study to apply the OPTIMA-ID prescribing criteria to the medication data of older adults with intellectual disability and the first to investigate prevalence of PIP in older adults with intellectual disability in the Irish population. The majority of OPTIMA-ID criteria are applicable to the data from Wave 5 of IDS-TILDA, and functions for all applicable criteria have been designed. A strength of this is the use of efficient and automated computational methods instead of reliance on manual data manipulation.

Another strength is that the sample population is representative of the national population of older adults with intellectual disabilities in Ireland. IDS-TILDA recruited a sample of adults aged ≥40, proportional to the Irish population in term of level of intellectual disability and residential circumstance. This provides the study with the advantage that PIP will be detected across a representative sample of residential circumstances, instead of one organisation or service type, as is common in existing studies assessing PIP in this population. ^47^ ^–^ ^49^ ^,^ ^57^ The inclusion of adults ≥40 reflects the reduced life expectancy of this population. ^5^ This study will demonstrate the usefulness of the tool and its potential for use in clinical practice to manage the comorbid conditions, polypharmacy and unique medicines management needs of this group.

### Limitations

While OPTIMA-ID has been developed for use in clinical practice, this study is a retrospective observational study without access to medical notes or medication reviews with participants or their carers beyond the interview and survey. Not every criterion can be applied to the dataset, and some criteria require the adoption of assumptions or generalisation. As the study does not consist of clinical medication reviews with the expertise of a pharmacist or other healthcare professional, what the algorithm detects as inappropriate may be appropriate for that individual. This means it is only possible to identify medicines that are “potentially” inappropriate in theory, and this method may overestimate or underestimate inappropriate prescribing in reality. The computational method for determining PIP is not intended to replace clinician judgement.

Another limitation is that the data used to apply the tool are self-reported, which may result in over or underreporting of PIP within the cohort. However, self-reported data regarding psychotropics from IDS-TILDA have recently been shown to be in good agreement with real prescribing records. ^58^

## Data Availability

Zenodo: Extended Data 1 (data operationalisation) and Extended Data 2 (code) in ‘Application of OPTIMA-ID to identify potentially inappropriate prescribing in a cohort of older adults with intellectual disability’, https://doi.org/10.5281/zenodo.20556000. ^53^ Data are available under the terms of Creative Commons Attribution 4.0 International (CC-BY 4.0). The output from the study investigating PIP prevalence will be reported using STrengthening the Reporting of OBservational studies in Epidemiology (STROBE) reporting guidelines for cohort studies and the follow up at Wave 6 with the corresponding guidelines for cohort studies. ^59^

## References

[1] Patja K Iivanainen M Vesala H : Life expectancy of people with intellectual disability: a 35-year follow-up study. *J. Intellect. Disabil. Res.* 2000 Oct 1;44(5):591–599. 10.1046/j.1365-2788.2000.00280.x 11079356

[2] Coppus AMW : People with intellectual disability: What do we know about adulthood and life expectancy?. *Dev. Disabil. Res. Rev.* 2013 Aug 1;18(1):6–16. 10.1002/ddrr.1123 23949824

[3] Heslop P Blair PS Fleming P : The Confidential Inquiry into premature deaths of people with intellectual disabilities in the UK: a population-based study. *Lancet.* 2014 Mar 8;383(9920):889–895. 10.1016/S0140-6736(13)62026-7 24332307

[4] Glover G Williams R Heslop P : Mortality in people with intellectual disabilities in England. *J. Intellect. Disabil. Res.* 2017;61(1):62–74. 10.1111/jir.12314 27489075

[5] McCarron M Swinburne J Burke E : Patterns of multimorbidity in an older population of persons with an intellectual disability: Results from the intellectual disability supplement to the Irish longitudinal study on aging (IDS-TILDA). *Res. Dev. Disabil.* 2013 Jan;34(1):521–527. 10.1016/j.ridd.2012.07.029 23085501

[6] Peklar J Kos M O’Dwyer M : Medication and supplement use in older people with and without intellectual disability: An observational, cross-sectional study. Forloni G, editor. *PLoS ONE.* 2017 Sep 6;12(9):e0184390. 10.1371/journal.pone.0184390 28877256 PMC5587307

[7] Hermans H Evenhuis HM : Multimorbidity in older adults with intellectual disabilities. *Res. Dev. Disabil.* 2014 Apr 1;35(4):776–783. 10.1016/j.ridd.2014.01.022 24529858

[8] O’Dwyer M Peklar J McCallion P : Factors associated with polypharmacy and excessive polypharmacy in older people with intellectual disability differ from the general population: a cross-sectional observational nationwide study. *BMJ Open.* 2016 Apr;6(4):e010505. 10.1136/bmjopen-2015-010505 27044582 PMC4823458

[9] Schoufour JD Oppewal A Maarl HJK van der : Multimorbidity and Polypharmacy Are Independently Associated With Mortality in Older People With Intellectual Disabilities: A 5-Year Follow-Up From the HA-ID Study. *Am. J. Intellect. Dev. Disabil.* 2018 Jan 1;123(1):72–82. 10.1352/1944-7558-123.1.72 29281324

[10] Zaal RJ Kaaij ADM van der Evenhuis HM : Prescription errors in older individuals with an intellectual disability: Prevalence and risk factors in the Healthy Ageing and Intellectual Disability Study. *Res. Dev. Disabil.* 2013 May 1;34(5):1656–1662. 10.1016/j.ridd.2013.02.005 23501585

[11] O’Connell J Henman MC McMahon N : Medication burden and frailty in older adults with intellectual disability: An observational cross-sectional study. *Pharmacoepidemiol. Drug Saf.* 2020;29(4):482–492. 10.1002/pds.4987 32134549

[12] Petropoulou E Skelton DA Finlayson J : Predicting Injuries in Adults With Intellectual Disabilities in Supported Living: Development and Validation of Risk Assessment Tools. 2026. 10.1111/jar.70253 42186697

[13] Mekonnen A Redley B Crawford K : Associations between hyper-polypharmacy and potentially inappropriate prescribing with clinical and functional outcomes in older adults. *Expert Opin. Drug Saf.* 2022 Jul 3;21(7):985–994. 10.1080/14740338.2022.2044786 35180833

[14] Moriarty F Hardy C Bennett K : Trends and interaction of polypharmacy and potentially inappropriate prescribing in primary care over 15 years in Ireland: a repeated cross-sectional study. *BMJ Open.* 2015 Sep 1;5(9):e008656. 10.1136/bmjopen-2015-008656 26384726 PMC4577876

[15] O’Dwyer M Maidment ID Bennett K : Association of anticholinergic burden with adverse effects in older people with intellectual disabilities: an observational cross-sectional study. *Br. J. Psychiatry.* 2016 Dec;209(6):504–510. 10.1192/bjp.bp.115.173971 27660331

[16] O’Connell J Burke É Mulryan N : Drug burden index to define the burden of medicines in older adults with intellectual disabilities: An observational cross-sectional study. *Brit J Clinical Pharma.* 2018 Mar;84(3):553–567. 10.1111/bcp.13479 29193284 PMC5809360

[17] Al Shuhaimi L Maidment ID Henman MC : Ten-Year Outcomes of Anticholinergic Use Among Older Adults With Intellectual Disability: Findings From the Intellectual Disability Supplement to the Irish Longitudinal Study on Ageing (IDS-TILDA). *J. Intellect. Disabil. Res.* 2025;69(11):1284–1294. 10.1111/jir.70018 40713937 PMC12576370

[18] Margallo-Lana ML Moore PB Kay DWK : Fifteen-year follow-up of 92 hospitalized adults with Down’s syndrome: incidence of cognitive decline, its relationship to age and neuropathology. *J. Intellect. Disabil. Res.* 2007 Jun;51(Pt. 6):463–477. 10.1111/j.1365-2788.2006.00902.x 17493029

[19] Schupf N Zigman W Kapell D : Early menopause in women with Down’s syndrome. *J. Intellect. Disabil. Res.* 1997 Jun;41(Pt 3):264–267. 10.1111/j.1365-2788.1997.tb00706.x 9219076

[20] Cosgrave MP Tyrrell J McCarron M : Age at onset of dementia and age of menopause in women with Down’s syndrome. *J. Intellect. Disabil. Res.* 1999 Dec;43(Pt 6):461–465. 10.1046/j.1365-2788.1999.00192.x 10622361

[21] Ejskjaer K Uldbjerg N Goldstein H : Menstrual profile and early menopause in women with Down syndrome aged 26-40 years. *J. Intellect. Develop. Disabil.* 2006 Sep;31(3):166–171. 10.1080/13668250600879222 16954095

[22] Winterhalder R : Psychopharmacological Issues. *Learning Disability and Other Intellectual Impairments.* John Wiley & Sons, Ltd;2008; p.165–77. 10.1002/9780470697863.ch9

[23] Sheehan R Horsfall L Strydom A : Movement side effects of antipsychotic drugs in adults with and without intellectual disability: UK population-based cohort study. *BMJ Open.* 2017 Aug 3;7(8):e017406. 10.1136/bmjopen-2017-017406 28775195 PMC5724123

[24] O’Dwyer C McCallion P Henman M : Prevalence and patterns of antipsychotic use and their associations with mental health and problem behaviours among older adults with intellectual disabilities. *J. Appl. Res. Intellect. Disabil.* 2019;32(4):981–993. 10.1111/jar.12591 31038275

[25] Sheehan R Hassiotis A Walters K : Mental illness, challenging behaviour, and psychotropic drug prescribing in people with intellectual disability: UK population based cohort study. *BMJ.* 2015 Sep 1;h4326. 10.1136/bmj.h4326 26330451 PMC4556752

[26] Brylewski J Duggan L : Antipsychotic medication for challenging behaviour in people with learning disability. *Cochrane Database of Systematic Reviews.* 2004 Jul 19 [cited 2025 Dec 20];2009:CD000377. 10.1002/14651858.CD000377.pub2/full 15266428 PMC12334078

[27] Kuijper G de Jonker J Hoge R : Structured Medication Review and Shared Decision-Making in Patients with Mild Intellectual Disabilities Who Use Psychotropic Medication. *Pharmacy.* 2026 Feb;14(1):5. 10.3390/pharmacy14010005 41562973 PMC12821519

[28] NICE: Medicines optimisation: the safe and effective use of medicines to enable the best possible outcomes. 2015 [cited 2025 Dec 17]. Reference Source

[29] Carollo M Crisafulli S Vitturi G : Clinical impact of medication review and deprescribing in older inpatients: A systematic review and meta-analysis. 10.1111/jgs.19035 38822740

[30] Riordan DO Walsh KA Galvin R : The effect of pharmacist-led interventions in optimising prescribing in older adults in primary care: A systematic review. *Sage Open Medicine.* 2016 Jan 1;4:2050312116652568. 10.1177/2050312116652568 27354917 PMC4910534

[31] Thayer N White S Islam J : Reducing risks associated with medicines and lifestyle in a residential care population with intellectual disabilities: evaluation of a pharmacy review initiative in England. *BMJ Open.* 2021 Aug 17;11(8):e046630. 10.1136/bmjopen-2020-046630 34404698 PMC8372807

[32] 2023 American Geriatrics Society Beers Criteria® Update Expert Panel: American Geriatrics Society 2023 updated AGS Beers Criteria® for potentially inappropriate medication use in older adults. *J. Am. Geriatr. Soc.* 2023;71(7):2052–2081. 10.1111/jgs.18372 37139824 PMC12478568

[33] O’Mahony D Cherubini A Guiteras AR : STOPP/START criteria for potentially inappropriate prescribing in older people: version 3. *Eur Geriatr Med.* 2023 Aug;14(4):625–632. 10.1007/s41999-023-00777-y 37256475 PMC10447584

[34] Doherty AS Moriarty F Boland F : Prevalence of potentially inappropriate prescribing in community-dwelling older adults: an application of STOPP/START version 3 to The Irish Longitudinal Study on Ageing (TILDA). *Eur Geriatr Med.* 2025;16(4):1389–1402. 10.1007/s41999-025-01201-3 40295430 PMC12378767

[35] O’Dwyer M McCallion P McCarron M : Medication use and potentially inappropriate prescribing in older adults with intellectual disabilities: a neglected area of research. *Therapeutic Advances in Drug Safety.* 2018 Sep;9(9):535–557. 10.1177/2042098618782785 30181861 PMC6116771

[36] O’Dwyer M McCallion P McCarron M : Measuring drug burden in older adults with intellectual disabilities: Critical issues for consideration in finding the optimal measure to improve safety of medicines use. *Expert Opin. Drug Saf.* 2020 Jun 2;19(6):649–652. 10.1080/14740338.2020.1751119 32241202

[37] Dimitrow MS Airaksinen MSA Kivelä SL : Comparison of Prescribing Criteria to Evaluate the Appropriateness of Drug Treatment in Individuals Aged 65 and Older: A Systematic Review. *J. Am. Geriatr. Soc.* 2011;59(8):1521–1530. 10.1111/j.1532-5415.2011.03497.x 21797829

[38] Lennox NG Kerr MP : Primary health care and people with an intellectual disability: the evidence base. *J. Intellect. Disabil. Res.* 1997;41(5):365–372. 10.1111/j.1365-2788.1997.tb00723.x 9373816

[39] O’Connell J Gorman A Burke É : OPTIMA-ID: development and validation of a medicine optimization tool for older adults with intellectual disability. *Expert. Rev. Clin. Pharmacol.* 2024 Sep;17(9):837–851. 10.1080/17512433.2024.2390913 39138862

[40] Linstone HA : *The Delphi method: techniques and applications.* Reading, Mass: Addison-Wesley; 3. pr ed 2002;620.

[41] Aging Brain Care: *The Anticholinergic Cognitive Burden Scale (2012 Update).* Indiana University of Aging Research ABP;2012 [cited 2026 Nov 4]. Reference Source

[42] Whaikaha - Ministry of Disabled People: *New Zealand Disability Strategy.* Te Kāwanatanga o Aotearoa - New Zealand Government;2016 Nov [cited 2025 Dec 1]. Reference Source

[43] Australian Government: 2024 update: Building a more inclusive Australia. Australia’s Disability Strategy 2021-2031. 2025 Jun [cited 2025 Dec 1]. Reference Source

[44] Department of Children, Disability and Equality: *National Human Rights Strategy for Disabled People 2025-2030.* Government of Ireland;2025 Sep [cited 2025 Jan 12]. Reference Source

[45] Working Group on Congregated Settings: *Time to Move On from Congregated Settings.* Health Service Executive;2011 Jun [cited 2025 Dec 1]. Reference Source

[46] Marquis S O’Leary R Marquis NE : A Rapid Mapping Review of Medication Burden in Adults with an Intellectual Disability: What We Know and What We Do Not Know. *DHPS.* 2026 Mar 11;18:1–24. 10.2147/DHPS.S558001 41853486 PMC12992441

[47] Zaal RJ Ebbers S Borms M : Medication review using a Systematic Tool to Reduce Inappropriate Prescribing (STRIP) in adults with an intellectual disability: A pilot study. *Res. Dev. Disabil.* 2016 Aug 1;55:132–142. 10.1016/j.ridd.2016.03.014 27065309

[48] Scheifes A Egberts TCG Stolker JJ : Structured Medication Review to Improve Pharmacotherapy in People with Intellectual Disability and Behavioural Problems. *Research Intellect Disabil.* 2016 Jul;29(4):346–355. 10.1111/jar.12183 25882186

[49] Lim AG Garriock J Moody I : Potentially inappropriate medicines for older adults with intellectual disability: Clinical implications from a medication audit. *Australas. J. Ageing.* 2021;40(3):e207–e214. 10.1111/ajag.12900 33523552

[50] Lonchampt S Gerber F Aubry JM : TOP-ID: a Delphi technique-guided development of a prescription and deprescription tool for adults with intellectual disabilities. *BMJ Open.* 2020 Nov 1;10(11):e039208. 10.1136/bmjopen-2020-039208 33148748 PMC7643515

[51] Jonker J Kuijper GM de Zuidema SU : Developing and testing of an assessment tool for appropriate psychotropic drug prescribing in people with intellectual disabilities. *Ther Adv Drug Saf.* 2025 Jul 8;16:20420986251342351. 10.1177/20420986251342351 40655471 PMC12246530

[52] R Core Team: *R: A Language and Environment for Statistical Computing.* Vienna, Austria: R Foundation for Statistical Computing;2026. Reference Source

[53] Ryan I : Application of OPTIMA-ID to identify potentially inappropriate prescribing in a cohort of older adults with intellectual disability. *Zenodo.* 2026. 10.5281/zenodo.20556000

[54] Wade DT Collin C : The Barthel ADL Index: a standard measure of physical disability?. *Int. Disabil. Stud.* 1988;10(2):64–67. 10.3109/09638288809164105 3042746

[55] Odalović M Gorman A Paul A : Psychotropic medicines’ prevalence, patterns and effects on cognitive and physical function in older adults with intellectual disability in Ireland: longitudinal cohort study, 2009–2020. *BJPsych Open.* 2024 Mar;10(2):e39. 10.1192/bjo.2023.607 38297892 PMC10897684

[56] Peduzzi P Concato J Kemper E : A simulation study of the number of events per variable in logistic regression analysis. *J. Clin. Epidemiol.* 1996 Dec;49(12):1373–1379. 10.1016/s0895-4356(96)00236-3 8970487

[57] Granas AG Halvorsen KH Wendelbo J : Interdisciplinary medication review to improve pharmacotherapy for patients with intellectual disabilities. *Int. J. Clin. Pharm.* 2019 Dec 1;41(6):1516–1525. 10.1007/s11096-019-00914-3 31729635

[58] Gorman A Odalović M McCallion P : Comparing self-report medication data from a longitudinal study on intellectual disability and national dispensing records. *J. Intellect. Disabil. Res.* 2025;69(1):103–111. 10.1111/jir.13192 39403989 PMC11621587

[59] Von Elm E Altman DG Egger M : The Strengthening the Reporting of Observational Studies in Epidemiology (STROBE) Statement: Guidelines for Reporting Observational Studies. *Ann. Intern. Med.* 2007 Oct 16;147(8):573–577. 10.7326/0003-4819-147-8-200710160-00010 17938396

