# Supplementary material for: A protocol for application of OPTIMA-ID to identify potentially inappropriate prescribing in a cohort of older adults with intellectual disability
Source: HRB Open Res. 2026 Jul 29;9:81. [Version 1] doi: 10.12688/hrbopenres.14510.1 (PMC13560664; doi:10.12688/hrbopenres.14510.1)
Supplement: Peer review history

# Reviewer report: Reviewer response for version 1

**DOI:** 10.21956/hrbopenres.15993.r57027

**Published:** 2026-08-19

**Authors:** Rory Sheehan

**Affiliations:** 1 King’s College London, London, UK

This paper describes the adaptation of the OPTIMA-ID prescribing criteria to the existing IDS-TILDA dataset through the development of a set of operationalised statements and their application to the dataset. The authors find that the majority of OPTIMA-ID criteria can be applied to the data, which will help to describe and measure potentially inappropriate prescribing in this group. The topic is important and the method described in the paper will be of benefit in future research.  

 The issues facing older adults with intellectual disability and rationale for the paper are well established in the introduction, including appropriate references and comparisons with the non-intellectually disabled population. There is a balanced discussion of the pros- and cons- of prescribed medication, including the concept of potentially inappropriate prescribing, medication optimisation, and the practical challenges in this group. Background related to the need and development of the OPTIMA-ID tool is adequately explained. The objectives of the study are clearly stated.  

 The methods are clear and the work draws on data collected from a pre-existing longitudinal study and programme of work, which is well-respected and has given rise to other important publications. Planned data analysis methods are described in detail and are appropriate and the results will have practical implications. 

 The dissemination plan follows a standard approach. A little more detail could be provided on how the PPI group have influenced the study to this point and the opportunities for people with lived experience to contribute going forward beyond consultation and being informed of the results. 

 The strengths and limitations are acknowledged, many of which are common to the IDS-TILDA study overall and the authors already identify the implications of these on the strength of the findings that will be generated.

Is the study design appropriate for the research question?

Yes

Is the rationale for, and objectives of, the study clearly described?

Yes

Are sufficient details of the methods provided to allow replication by others?

Yes

Are the datasets clearly presented in a useable and accessible format?

Yes

Reviewer Expertise:

Intellectual disability and neurodevelopmental disorders

I confirm that I have read this submission and believe that I have an appropriate level of expertise to confirm that it is of an acceptable scientific standard.

# Reviewer report: Reviewer response for version 1

**DOI:** 10.21956/hrbopenres.15993.r57024

**Published:** 2026-08-14

**Authors:** Steven R. Erickson

**Affiliations:** 1 University of Michigan, Ann Arbor, USA

*Is the rationale for, and objectives of, the study clearly described?*

 The investigators write a compelling and informative rationale for the study.  They provide an extensive background section that is written well.  When the reader has finished reading this section, they will have a clear understanding of problem which this study addresses, the instruments that currently exist, and the rational for creating a new instrument focused on older adults with intellectual disability.  This section further discusses and documents the development of the OPTIMA-ID which is important to understand in the Methods section. 

*Is the study design appropriate for the research question?*

 The study design is well suited for this study.  The dataset, the sample (inclusion criteria) is defined and appropriate.  Data operationalization is defined and understandable. There are a few specific comments that may improve the full understanding of the data.  1.) Falls and Barthel index are mentioned as an outcomes variables near the end of the Analysis section, but are not listed in the objectives of the study.  Consistency in identification of important outcomes would be helpful.  2.) Additional covariates of interest may be residential setting (mentioned as data in the dataset) and prescriber type (not explicitly mentioned but may be useful).  3.) Multimorbidity is mentioned as a covariate. This is indeed important to control for.  The measure was developed using the same dataset used for this study, and is based on the Charlson comorbidity index, with 2 additional conditions added that are common for people with intellectual disability.  The Charlson index was developed to predict mortality during hospitalization and has been used extensively to control for multimorbidity in many other types of outcomes.  Have the investigators considered using a more contemporary index such as the Elixhauser index?  It is a strong predictor not only for mortality, but for healthcare utilization, health-related quality of life, and other important outcomes.  4.) The investigators mention in the very last sentence of the Analysis section that data may be censured to safeguard the identity of study participants.  It may be helpful to add more explanation of the decision rule guiding this potential decision. 

*Are sufficient details of the methods provided to allow replication by others?*

 The protocol, and especially the analysis section, provide adequate information so that independent researchers would be able to replicate the study.  Information on operationalization of the instrument is provided in references. 

*Are the datasets clearly presented in a useable and accessible format?*

 The dataset is appropriate to use for this study based on description given in the manuscript.  The Sources of Data section provides a clear description of the dataset.  The investigators may want to add a few sentences about the historical development of the dataset and provide key references for the reader to follow.

Is the study design appropriate for the research question?

Yes

Is the rationale for, and objectives of, the study clearly described?

Yes

Are sufficient details of the methods provided to allow replication by others?

Yes

Are the datasets clearly presented in a useable and accessible format?

Yes

Reviewer Expertise:

Health services research on medication management for persons with disabilities

I confirm that I have read this submission and believe that I have an appropriate level of expertise to confirm that it is of an acceptable scientific standard.
